# Arm swing deviations in patients with Parkinson’s disease at different gait velocities

**DOI:** 10.1007/s00702-023-02619-4

**Published:** 2023-03-14

**Authors:** Stefan Mainka, Maximilian Lauermann, Georg Ebersbach

**Affiliations:** 1 Movement Disorder Clinic, Parkinsonklinik, Str. n. Fichtenwalde 16, 14547 Beelitz-Heilstätten, Germany; 2 Faculty of Human Sciences, University of Potsdam, Potsdam, Germany

**Keywords:** Walking, Arm swing, Gait velocity, Parkinson’s disease

## Abstract

Asymmetry of arm swing (AS) has been described as a characteristic of normal physiological gait. In patients with Parkinson’s disease (PWPD), a one-sided reduction of AS can occur already as a prodromal symptom. There is limited evidence regarding AS in PWPD, but a growing interest in AS as a focus of exercise therapy. The differences of AS between 32 healthy subjects (HS) and 36 mildly-to-moderately impaired PWPD were assessed in overground walking at various gait speeds. Assessments were carried out with a sensor-based gait measurement system over a 40 m walk in very slow, slow, preferred, fast, and very fast gait speed. Longitudinal and AS kinematics were compared with ANOVA function and regression analysis. PWPD exhibited a one-sided reduction of AS compared to HS at normal, fast, and very fast walking. AS coordination, representing the timing of reciprocity of right and left AS, was reduced in PWPD in very slow and normal walking. With respect to leg movements, PWPD exhibited an increase in stride time variability in very slow gait. There were no group differences for cadence, stride length, and gait velocity. This study informs about the kinematics of AS at various gait velocities ranging from very slow to very fast in mildly-to-moderately impaired PWPD. Reduced one-sided AS can be considered as a very early sign of parkinsonian gait disturbance that precedes alterations of locomotive leg movements and improves at faster gait speeds.

## Introduction

Walking is the most fundamental motor function in human beings and has a large impact on autonomy, participation, and quality of life (Breek et al. 2002). Parkinson’s disease (PD) is a neurodegenerative disease, leading to slower and smaller movements (bradykinesia) (Marsden 1982). Bradykinesia also affects overground walking. Zanardi et al. reviewed data from 68 studies evaluating overground walking at self-selected speed in patients with PD (PWPD) (*n* = 1425) and healthy subjects (HS) (*n* = 1402). They found reduced walking speed, stride length, swing time, and hip excursion in PWPD, as well as an increased cadence and double support time (Zanardi et al. 2021). These gait alterations in PWPD are associated with a significant reduction of daily movement in the course of the disease and can increase the risk of falls (Boonstra et al. 2008).

Arm swing (AS) is an essential component of human gait that leads to a notable reduction of energetic costs (Meyns et al. 2013). Even though AS is mainly passive, muscle activity is needed to maintain sufficient AS (Goudriaan et al. 2014). The empiric data on upper extremity gait kinematics in PWPD and their functional impact are comparatively limited, but have received more and more attention in the past years. Nonetheless, an increased asymmetry with a one-sided reduction of AS that is pronounced in the retroverse component has been described (Lewek et al. 2010; Huang et al. 2012; Roggendorf et al. 2012; Ospina et al. 2018) and has even been suggested as a prodromal marker (Mirelman et al. 2016) that is responsive to dopaminergic medication (Warmerdam et al. 2021). The one-sided reduction of AS can lead to a higher risk of falls in PWPD (Wood et al. 2002). This contributes to the fact that AS has recently become a focus for motor training (Thompson et al. 2017; Zampier et al. 2018; Mainka et al. 2021). There is a great extent of AS asymmetry in normal, unimpaired walking, that is not related to handedness and often manifests in a larger AS on the left side compared to the right (Kuhtz-Buschbeck et al. 2008; Killeen et al. 2018). Killeen et al. found a mean non-directional asymmetry index (ndASI) of 39.5 ± 21.8 in overground walking of HS in their sample (N = 334) (Killeen et al. 2018). On the other hand, Plate et al. found lower AS asymmetry on the treadmill at 3 (32.7 ± 22.0) and 4 km/h (27.9 ± 20.8). Due to a comparison with PWPD here, an ndASI above 50 was considered pathological (Plate et al. 2015). Recent developments in sensor-based gait analysis systems (Morris et al. 2019; Flachenecker et al. 2020; Di Lazzaro et al. 2021) enable researchers to evaluate habitual overground walking in a larger area with fewer spatial and optical constraints compared to those in the treadmill trials in laboratory conditions.

To our knowledge, a direct comparison of AS measures in overground walking between HS und PWPD at other than the preferred and maximum gait speed has not yet been studied. Therefore, differences in AS between HS and PWPD during overground walking at various walking speeds were assessed in the present trial.

## Materials and methods

The study was registered with the German clinical trials register (DRKS00022049). We included patients diagnosed with PD according to UK Brain Bank criteria. These patients met the following criteria: age 40–79 years, ability to walk safely without use of walking aids, ability to change gait speed from very slow to very fast, ability to perform testing without fatigue based on previous experience of the participant, no further gait disorder that was not related to PD, no troublesome dyskinesia in on-state of dopaminergic medication (corresponding to score 0 in UPDRS item IV.33), no camptocormia or Pisa-syndrome > 20°, no festination of gait with propulsive gait pattern and insufficient heel strike, no freezing of gait during straight walking episodes during the last 6 months, and no signs or history of cognitive impairment (corresponding to score 0 in UPDRS item I.1.).

We recruited age-matched HS with good walking ability as a control group. Participants were instrumented for gait analysis with six inertial measurement units (IMU; Mobility Lab, APDM, USA) by applying sensors at the fifth lumbar vertebrae (5LV) and sternum and one sensor fixed to each metatarsus and wrist dorsally with a Velcro™ strap.

Testing for PWPD included the UPDRS III motor score and was performed in the ON-state of dopaminergic medication. Assessments comprised six straight forward 40 m walks along a wide corridor. Two preparatory trials at preferred speed were carried out before these six test walks were performed to enable participants to become familiar with the procedure. After this, the participants were asked to walk at very slow, slow, preferred, fast, and very fast speeds. This was done in the following fixed order: 1 preferred, 2 slow, 3 fast, 4 preferred, 5 very slow, and 6 very fast. The data from trial 2 to 5 were taken for the statistical analysis.

The 4.8. × 1.3 × 3.6 cm IMUs weigh 22 g and measure with a sampling frequency of 128 Hz.

Cadence, gait velocity, stride length (average of right- and left-side values), and range of motion (ROM) in the sagittal (rotation) plane of the 5LV and the sternum were computed by APDM Mobility Lab (ML) system (version 2) (Morris et al. 2019). The algorithm by Warmerdam and colleagues (Warmerdam et al. 2020) was used to compute the AS parameters ROM, peak angular velocity (PAV), and regularity, which represents the similarity of neighboring swings based on the angular velocity and coordination, representing the timing of reciprocity of right and left AS. AS ROM values were used to calculate the non-directional asymmetry indices with the formula

$${\text{ndASI}} = {\text{ABS}}\left( {\frac{L - R}{{\max \left( {L,R} \right)}}} \right) \times 100,$$

where *R* and *L* represent right and left AS ROM mean values (Killeen et al. 2018).

The statistical analysis was carried out with R (version 4.0.3). To check for differences between the five walking modi and the two cohorts, linear mixed-effects regression models were run with the R package nlme using walking modus and group as fixed effect factors and random effect intercept for the subject. The model included also the interaction between walking modus and group. Main effects were checked using the anova function of R.

If significant main effects were found, post hoc comparisons were carried out with R package emmeans, adjusting the p values using the procedure of Benjamini and Hochberg to control for the false discovery rate at a level of 0.05.

For target parameters AS ROM, AS PAV, stride, and cadence, we also ran a linear mixed-effects model treating speed as continuous predictor. For the AS parameters, we compared averaged values from the right and left sides of the HS to the more affected side of the PWPD. The model included intercept and slope for each group separately and random intercept and slope for each subject. Differences in intercept and slope were tested for each group.

Two sub-group analyses were performed for the AS ROM of the less swinging side and AS asymmetry in PWPD. In the first sub-group analysis, patients with shorter durations of the disease (≤ 5 years) were compared to cases with longer durations (> 5 years). Second, following the formula used by Stebbins (Stebbins et al. 2013), we sorted the PWPD into the tremor dominant (TD) and the postural instability/gait difficulty (PIGD) subtypes.

## Results

We recruited 32 HS and 36 PWPD that did not differ in their anthropometric characteristics (Table 1). All PWPD were under dopaminergic medication. Two of these patients had ongoing deep brain stimulation. Both HS and PWPD were able to regulate their gait tempi as requested within the experimental setting. There were no significant differences in cadence, gait velocity, and stride between HS and PWPD across all five walking speeds (Table 2). PWPD exhibited an increase in stride time variability in the very slow condition.

**Table 1:** Subject data of healthy subjects and individuals with Parkinson’s disease (PD)

	Healthy	PD
Number	32	36
Age [years]	64.5 ± 9.0	61.7 ± 7.3
Gender (F/M)	17 / 15	19 / 17
Height [cm]	172.1 ± 10.3	171.8 ± 10.8
Weight [kg]	78.8 ± 13.9	76.6 ± 16.2
Disease duration [years]	N/A	4.8 ± 3.4
Hoehn & Yahr stage	N/A	2.0 ± 0.6
UPDRS III motor score	N/A	17.6 ± 7.7
Levodopa equivalent daily dose [mg]	N/A	715.5 ± 265.6

*Significant difference between groups with *p* values < 0.05

**Table 2:** Kinematic measurements (mean ± standard deviation) for 32 healthy subjects and 36 patients with PD at five different gait velocities

	Very slow		Slow		Preferred		Fast		Very fast	
	Healthy	PD	Healthy	PD	Healthy	PD	Healthy	PD	Healthy	PD
Cadence [spm]	82.8 ± 14.8	82.2 ± 13.7	94.0 ± 14.9	96.6 ± 9.0	111.1 ± 16.7	114.0 ± 6.9	125.1 ± 19.8	125.8 ± 6.8	135.6 ± 21.3	137.4 ± 9.0
Gait velocity [m/s]	0.74 ± 0.18	0.67 ± 0.18	0.95 ± 0.16	0.92 ± 0.14	1.28 ± 0.13	1.26 ± 0.12	1.59 ± 0.13	1.56 ± 0.13	1.80 ± 0.15	1.79 ± 0.15
Stride [m]	1.03 ± 0.16	0.97 ± 0.15	1.17 ± 0.15	1.13 ± 0.11	1.34 ± 0.12	1.33 ± 0.12	1.48 ± 0.13	1.49 ± 0.13	1.55 ± 0.16	1.56 ± 0.14
Stride time variability [%] #	3.4 ± 1.5	**4.3 ± 1.9**	2.4 ± 1.0	2.6 ± 1.2	1.7 ± 0.8	1.4 ± 0.5	1.7 ± 0.6	1.7 ± 0.5	1.9 ± 0.9	2.0 ± 0.9
Pelvis ROM [°]	4.2 ± 1.3	4.2 ± 1.4	4.6 ± 1.7	4.5 ± 1.5	5.0 ± 1.6	5.5 ± 2.1	5.8 ± 1.9	7.1 ± 4.6	6.7 ± 2.3	7.6 ± 4.1
Sternum ROM [°]	4.0 ± 1.2	3.7 ± 1.2	4.0 ± 1.1	3.8 ± 1.1	4.2 ± 1.4	4.2 ± 1.2	4.9 ± 1.6	4.7 ± 1.3	5.4 ± 1.2	5.7 ± 1.6
AS^a^ ROM^b^ [°] less^c^ *#	18.5 ± 9.6	11.8 ± 8.7	24.2 ± 11.0	15.5 ± 8.8	46.9 ± 18.6	**28.8 ± 17.8**	65.9 ± 19.5	**45.4 ± 24.7**	79.5 ± 24.4	**61.1 ± 29.9**
AS ROM [°] stronger^d^	24.8 ± 12.0	21.7 ± 11.8	33.0 ± 12.2	30.9 ± 13.9	58.1 ± 19.1	53.7 ± 18.2	77.9 ± 18.5	73.8 ± 20.1	92.8 ± 21.5	89.8 ± 19.2
AS ROM ndASI^e^ (0–100)*#	24.8 ± 18.3	**41.6 ± 26.8**	26.6 ± 18.8	**42.7 ± 26.8**	19.5 ± 14.5	**44.5 ± 27.2**	15.6 ± 14.9	**39.3 ± 24.3**	14.9 ± 15.8	**34.0 ± 25.5**
AS peak angular velocity [°/s] less*#	70.9 ± 37.8	46.6 ± 30.1	91.3 ± 39.9	60.6 ± 33.0	159.8 ± 63.1	**101.6 ± 57.4**	219.2 ± 67.2	**152.4 ± 76.0**	267.2 ± 79.1	**206.2 ± 95.1**
AS peak angular velocity [°/s] stronger	94.1 ± 46.2	81.0 ± 40.9	122.3 ± 46.4	114.0 ± 48.0	194.8 ± 63.5	180.1 ± 59.3	258.3 ± 65.4	245.0 ± 67.0	309.9 ± 67.0	303.2 ± 64.6
AS regularity (0–1) less	0.82 ± 0.07	0.79 ± 0.16	0.88 ± 0.06	0.85 ± 0.09	0.93 ± 0.02	0.90 ± 0.07	0.95 ± 0.02	0.92 ± 0.06	0.95 ± 0.01	0.92 ± 0.08
AS regularity (0–1) stronger	0.84 ± 0.08	0.85 ± 0.08	0.89 ± 0.04	0.90 ± 0.06	0.94 ± 0.02	0.94 ± 0.02	0.95 ± 0.02	0.96 ± 0.02	0.95 ± 0.02	0.96 ± 0.01
AS coordination of arms (0–1)*	0.69 ± 0.09	**0.63 ± 0.11**	0.73 ± 0.11	0.69 ± 0.10	0.89 ± 0.07	**0.81 ± 0.12**	0.93 ± 0.03	0.88 ± 0.09	0.94 ± 0.03	0.90 ± 0.09

*Significant difference between groups

^#^Significant interaction between group and speed

^a^Arm swing

^b^Range of motion

^c^Less swinging side

^d^Stronger swinging side

^e^Non-directional asymmetry index

Bold: significant difference to healthy subjects, with *p* values < 0.05

We observed significant differences between both groups in AS. Subjects with PD exhibited a lower mean ROM and peak angular velocity in normal, fast, and very fast walking. This was mainly caused by decreases of the less swinging arm, while the stronger swinging side showed only a minor and non-significant decrease (Table 2).

Consequently, we found significantly higher asymmetry of AS in the PD group throughout. For the PWPD, the non-directional asymmetry index (ndASI) of AS was lowest during very fast walking which resembles a significant improvement compared to normal walking (*p* = 0.002).

The AS coordination as an index for reciprocal timing between right and left side showed significant decreases for PWPD in very slow and normal walking. This measure improved for both HS and PWPD with increasing velocity. The PD group reached values close to those of HS in fast and very fast walking. This increase was significant compared to normal walking (*p* < 0.001). The regularity of AS, a measure of similarity of consecutive ASs, did not differ between the two groups. There were no significant differences in pelvis and sternum rotation.

**Fig. 1:**
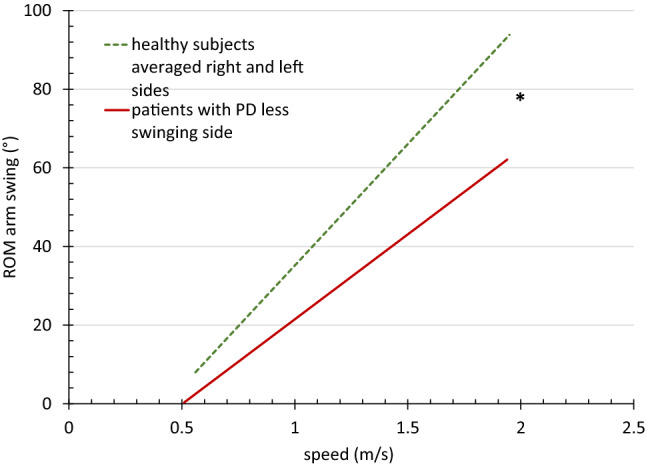
Regression line of arm swing range of motion values related to speed for 32 healthy subjects and 36 patients with PD, *significant difference

**Fig. 2:**
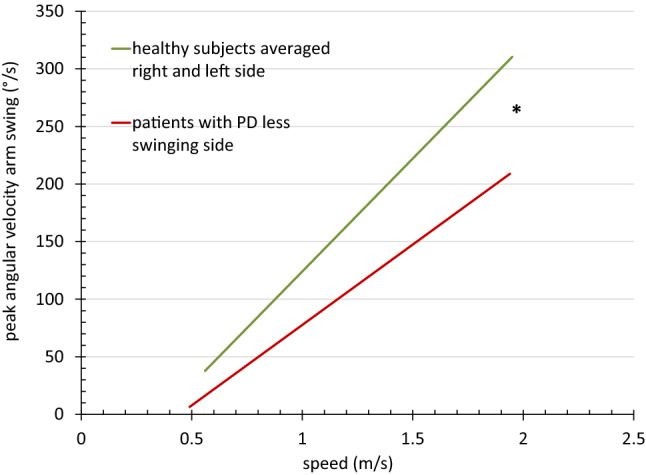
Regression line of arm swing peak angular velocity values related to speed for 32 healthy subjects and 36 patients with PD, *significant difference

**Fig. 3:**
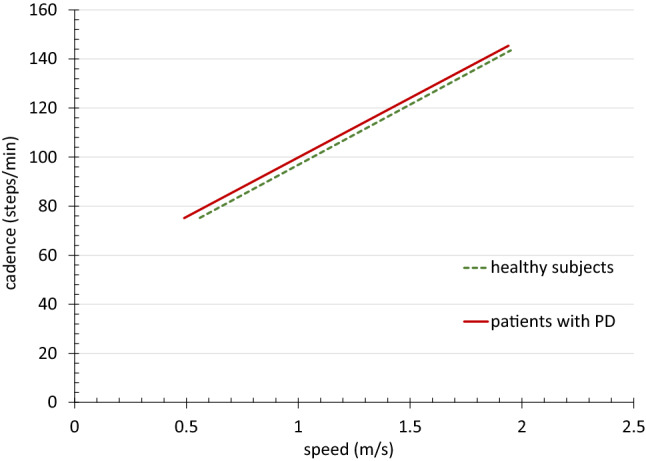
Regression line of cadence values related to speed for 32 healthy subjects and 36 patients with PD

**Fig. 4:**
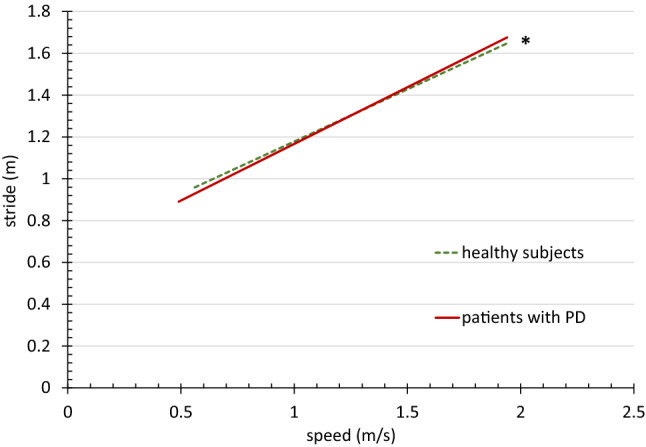
Regression line of stride length values related to speed for 32 healthy subjects and 36 patients with PD, *significant difference

In the regression analyses, the AS values narrowly missed significance for the difference in intercept (with *p* = 0.052 for ROM, and *p* = 0.055 for PAV) but showed a significant difference in slope (with *p* < 0.0001 for both) (Figs. 1, 2). We observed no statistical differences for cadence (with *p* = 0.159 for intercept and *p* = 0.780 for slope) (Fig. 3). We found significant differences for intercept (*p* = 0.015) and slope (*p* = 0.018) in the stride values (Fig. 4). This difference can be attributed to slightly lower values of the PWPD in very slow and slow walking.

In the sub-group analysis between 14 PWPD with shorter and 22 with longer durations of the disease, no significant differences in AS amplitude or asymmetry were observed. The symptom phenotype analysis revealed 14 subjects each from TD and the PIGD subtype, and 8 that were classified as intermediate. No significant differences in AS amplitude or asymmetry were observed between the two phenotypes.

## Discussion

To our knowledge, this is the first direct comparison of AS in overground walking of PWPD and HS at different gait speeds ranging from very slow to very fast. Even though there was no accompanying external cueing, PWPD were able to modify their gait velocity to the same extent as the HS. Despite the absence of lower limb differences, we observed a profound one-sided reduction of AS during overground walking in PWPD when compared to HS. This was reflected in lower values for ROM and PAV for PWPD in normal, fast, and very fast walking. Although at a lower level than the HS, PWPD had an automatic and significant increase in AS when walking faster. Larger AS at higher velocity was associated with increased bilateral phasic symmetry suggesting improved AS bilateral coordination. We observed normal AS values on the stronger swinging side. These results are in line with previous works (Huang et al. 2012; Roggendorf et al. 2012; Mirelman et al. 2016; Ospina et al. 2018).

It has been suggested to use AS asymmetry as a diagnostic factor for PD (Lewek et al. 2010). According to our data, the preferred walking condition would be the most informative source here. Looking at our sample of HS, there was only one subject with an ndASI > 40. This outlier had an exceptional high ndASI of 66.8. In comparison, 22 out of 36 subjects of the PD cohort showed an ndASI > 40 in which the proportion of subjects above that cutoff was higher for the TD subtype (10 out of 14) when compared to the PIGD subtype (7 out of 14). Therefore, we propose that for the preferred walking condition on even surfaces, an ndASI > 40 could serve as an additional diagnostic marker for PD. The TD sub-group and the short disease duration sub-group showed numerically higher AS asymmetry values throughout all gait tempi, but these differences did not reach statistical significance. Thus, for our sample, AS occurred to be more informative and clinically relevant than the lower limb parameters.

We did not find statistical differences in cadence, stride, and gait velocity in our sample that was comparable with respect to age, disease duration, Hoehn & Yahr stage, and UPDRS to the evaluated trials of the systematic review (Zanardi et al. 2021). The normal lower limb measurements corroborate the mild impairment in our study cohort. In addition, walking distance likely played a role in this result. In the systematic review of Zanardi, the mean walking distance in the overground walking trails was 11.9 ± 12.5 m, which is much shorter that our walkway of 40 m (Zanardi et al. 2021). Last but not least, we were able to test within a broad (3.2 m) floor, where there was no wall or boundary toward the end the of test track. This might have led to less interference with visuo-cognitive deficits during walking (Hill et al. 2016).

In human locomotion, AS has been shown to reduce the vertical ground reaction moment and oxygen consumption when compared to walking with constrained arms (Meyns et al. 2013). This may be explained by the reciprocity of AS in relation to the periodic vertical lifting motion during gait. If the arm is looked at as a pendulum, it would strive to take on its natural frequency according to Thomson’s oscillation equation. In this physical model, the system would operate at optimum if the step frequency is in resonance with the natural frequency of AS. In this mechanism, both arms reciprocally counterbalance and facilitate the lifting work of the body at each step (Bruening 2020). When looking at the PWPD, locomotion can be assumed to have higher energetic cost due to the one-sided reduction of AS even when the temporal parameters of leg movements are comparable to those of HS.

The strong and disproportionate increase of AS in PWPD on the more affected side in faster walking conditions led to significant improvements in AS symmetry and also in reciprocal coordination. It has been suggested that improving interlimb coordination between lower and upper extremities during walking might lead to a normalization of angular momentum and decrease energy expenditure (Meyns et al. 2013). There is evidence that an enforced AS can aid gait stability in HS (Wu et al. 2016). Additionally, it has been shown that the enforcement of AS leads to optimization of lower limb parameters in PWPD (Zampier et al. 2018; Mainka et al. 2021).

The increase of stride time variability during very slow walking prompts further clinical investigation as this feature is associated with falling in later stages (Schaafsma et al. 2003). Rhythmic auditory stimulation (RAS) through music stabilizes gait rhythmicity and has proven to be effective in preventing falls when step frequency is gradually increased (McIntosh et al. 1997; Thaut et al. 2019). Our data suggest evaluating stride time variability at slow gait velocities and may be an implication of the effectiveness of RAS at slower paces.

There are some limitations to our study: 1. Even though the assessed walking distance was longer than in other studies, 40 m is still fairly short and may not be sufficient to capture timing-sensitive features, such as stride time variability and AS coordination (Hollman et al. 2010). 2. The experimental setting with verbal instruction, start signal and sensors worn at four limbs and trunk is quite artificial and may affect PWPD more than HS. 3. In this study, we excluded PWPD with advanced parkinsonian gait disturbance, such as severe deviations of the trunk, festination of gait with propulsive gait pattern, and freezing of gait during straight walking. These features can be assumed to have a significant impact on gait parameters that is not conveyed by the present study.

Comparing ON- and OFF-state performance in this experimental setting would allow to further clarify the effect of dopaminergic medication on gait kinematics in PWPD. Furthermore, future studies should assess PWPD in the Hoehn & Yahr stages 2 and 3 to evaluate the impact of the TD and PIGD subtype on AS kinematics in the advanced course of the disease.

In sum, this study demonstrates the ability of mildly-to-moderately impaired PWPD to modify their gait speeds and cadence to the same extent as age-matched healthy individuals, despite considerable deficits in one-sided AS. Our data suggest that the improvement of AS in PWPD at higher gait velocities along with the reduction in AS symmetry may be a noteworthy focus of exercise therapy that should be considered early in the course of the disease.

## Data Availability

The datasets generated during and/or analyzed during the current study are available from the corresponding author on reasonable request.

## References

[CR1] Boonstra TA, Van Der Kooij H, Munneke M, Bloem BR (2008). Gait disorders and balance disturbances in Parkinson’s disease: clinical update and pathophysiology. Curr Opin Neurol.

[CR2] Breek JC, Hamming JF, De Vries J (2002). The impact of walking impairment, cardiovascular risk factors, and comorbidity on quality of life in patients with intermittent claudication. J Vasc Surg.

[CR3] Bruening HG (2020) Schrittfrequenz und Schwingungsweite der Arme beim Gehen. unpublished working paper

[CR4] Di Lazzaro G, Ricci M, Saggio G (2021). Technology-based therapy-response and prognostic biomarkers in a prospective study of a de novo Parkinson’s disease cohort. Npj Park Dis.

[CR5] Flachenecker F, Gaßner H, Hannik J (2020). Objective sensor-based gait measures reflect motor impairment in multiple sclerosis patients: Reliability and clinical validation of a wearable sensor device. Mult Scler Relat Disord.

[CR6] Goudriaan M, Jonkers I, van Dieen JH, Bruijn SM (2014). Arm swing in human walking: what is their drive?. Gait Posture.

[CR7] Hill E, Stuart S, Lord S (2016). Vision, visuo-cognition and postural control in Parkinson’s disease: an associative pilot study. Gait Posture.

[CR8] Hollman JH, Childs KB, McNeil ML (2010). Number of strides required for reliable measurements of pace, rhythm and variability parameters of gait during normal and dual task walking in older individuals. Gait Posture.

[CR9] Huang X, Mahoney JM, Lewis MM (2012). Both coordination and symmetry of arm swing are reduced in Parkinson’s disease. Gait Posture.

[CR10] Killeen T, Elshehabi M, Filli L (2018). Arm swing asymmetry in overground walking. Sci Rep.

[CR11] Kuhtz-Buschbeck JP, Brockmann K, Gilster R (2008). Asymmetry of arm-swing not related to handedness. Gait Posture.

[CR12] Lewek MD, Poole R, Johnson J (2010). Arm swing magnitude and asymmetry during gait in the early stages of Parkinson’s disease. Gait Posture.

[CR13] Mainka S, Schroll A, Warmerdam E (2021). The power of musification: sensor-based music feedback improves arm swing in Parkinson’s disease. Mov Disord Clin Pract.

[CR14] Marsden CD (1982). The mysterious motor function of the basal ganglia: the Robert Wartenberg Lecture. Neurology.

[CR15] McIntosh GC, Brown SH, Rice RR, Thaut MH (1997). Rhythmic auditory-motor facilitation of gait patterns in patients with Parkinson’s disease. J Neurol Neurosurg Psychiatry.

[CR16] Meyns P, Bruijn SM, Duysens J (2013). The how and why of arm swing during human walking. Gait Posture.

[CR17] Mirelman A, Bernad-Elazari H, Thaler A (2016). Arm swing as a potential new prodromal marker of Parkinson’s disease. Mov Disord.

[CR18] Morris R, Stuart S, Mcbarron G (2019). Validity of mobility lab (version 2) for gait assessment in young adults, older adults and Parkinson’s disease. Physiol Meas.

[CR19] Ospina BM, Chaparro JAV, Paredes JDA (2018). Objective arm swing analysis in early-stage Parkinson’s disease using an RGB-D Camera (Kinect ®). J Parkinsons Dis.

[CR20] Plate A, Sedunko D, Pelykh O (2015). Normative data for arm swing asymmetry: how (a)symmetrical are we?. Gait Posture.

[CR21] Roggendorf J, Chen S, Baudrexel S (2012). Arm swing asymmetry in Parkinson’s disease measured with ultrasound based motion analysis during treadmill gait. Gait Posture.

[CR22] Schaafsma JD, Giladi N, Balash Y (2003). Gait dynamics in Parkinson’s disease: relationship to Parkinsonian features, falls and response to levodopa. J Neurol Sci.

[CR23] Stebbins GT, Goetz CG, Burn DJ (2013). How to identify tremor dominant and postural instability/gait difficulty groups with the movement disorder society unified Parkinson’s disease rating scale: comparison with the unified Parkinson’s disease rating scale. Mov Disord.

[CR24] Thaut MH, Rice RR, Braun Janzen T (2019). Rhythmic auditory stimulation for reduction of falls in Parkinson’s disease: a randomized controlled study. Clin Rehabil.

[CR25] Thompson E, Agada P, Wright WG (2017). Spatiotemporal gait changes with use of an arm swing cueing device in people with Parkinson’s disease. Gait Posture.

[CR26] Warmerdam E, Romijnders R, Welzel J (2020). Quantification of arm swing during walking in healthy adults and Parkinson’s disease patients: Wearable sensor-based algorithm development and validation. Sensors (switzerland).

[CR27] Warmerdam E, Romijnders R, Hansen C (2021). Arm swing responsiveness to dopaminergic medication in Parkinson’s disease depends on task complexity. Npj Park Dis.

[CR28] Wood BH, Bilclough JA, Bowron A (2002). Incidence and prediction of falls in Parkinson’s disease: a prospective multidisciplinary study. J Neurol Neurosurg Psychiatry.

[CR29] Wu Y, Li Y, Liu AM (2016). Effect of active arm swing to local dynamic stability during walking. Hum Mov Sci.

[CR30] Zampier VC, Vitório R, Beretta VS (2018). Gait bradykinesia and hypometria decrease as arm swing frequency and amplitude increase. Neurosci Lett.

[CR31] Zanardi APJ, da Silva ES, Costa RR (2021). Gait parameters of Parkinson’s disease compared with healthy controls: a systematic review and meta-analysis. Sci Rep.

